# Near-Membrane Dynamics and Capture of TRPM8 Channels within Transient Confinement Domains

**DOI:** 10.1371/journal.pone.0013290

**Published:** 2010-10-11

**Authors:** Luis A. Veliz, Carlos A. Toro, Juan P. Vivar, Luis A. Arias, Jenifer Villegas, Maite A. Castro, Sebastian Brauchi

**Affiliations:** 1 Instituto de Fisiologia, Facultad de Medicina, Universidad Austral de Chile, Campus Isla Teja, Valdivia, Chile; 2 Instituto de Bioquimica, Facultad de Ciencias, Universidad Austral de Chile, Campus Isla Teja, Valdivia, Chile; 3 Instituto de Ingenieria Informatica, Facultad de Ciencias de la Ingenieria, Universidad Austral de Chile, Campus Isla Teja, Valdivia, Chile; Harvard Medical School, United States of America

## Abstract

**Background:**

The cold and menthol receptor, TRPM8, is a non-selective cation channel expressed in a subset of peripheral neurons that is responsible for neuronal detection of environmental cold stimuli. It was previously shown that members of the transient receptor potential (TRP) family of ion channels are translocated toward the plasma membrane (PM) in response to agonist stimulation. Because the spatial and temporal dynamics of cold receptor cell-surface residence may determine neuronal activity, we hypothesized that the movement of TRPM8 to and from the PM might be a regulated process. Single particle tracking (SPT) is a useful tool for probing the organization and dynamics of protein constituents in the plasma membrane.

**Methodology/Principal Findings:**

We used SPT to study the receptor dynamics and describe membrane/near-membrane behavior of particles containing TRPM8-EGFP in transfected HEK-293T and F-11 cells. Cells were imaged using total internal reflection fluorescence (TIRF) microscopy and the 2D and 3D trajectories of TRPM8 molecules were calculated by analyzing mean-square particle displacement against time. Four characteristic types of motion were observed: stationary mode, simple Brownian diffusion, directed motion, and confined diffusion. In the absence of cold or menthol to activate the channel, most TRPM8 particles move in network covering the PM, periodically lingering for 2–8 s in confined microdomains of about 800 nm radius. Removing cholesterol with methyl-beta-cyclodextrin (MβCD) stabilizes TRPM8 motion in the PM and is correlated with larger TRPM8 current amplitude that results from an increase in the number of available channels without a change in open probability.

**Conclusions/Significance:**

These results reveal a novel mechanism for regulating TRPM8 channel activity, and suggest that PM dynamics may play an important role in controlling electrical activity in cold-sensitive neurons.

## Introduction

Transient Receptor Potential (TRP) ion channels are cation-selective and calcium-permeable channels that contribute to a variety of sensory processes [1], [2]. Mammals express a subset of thermosensitive TRP channels, including the cold-activated TRPM8, that mediate sensation of environmental temperature [3]. TRPM8 activity is increased by cooling below ∼22°C, application of menthol, and changes in membrane potential [4], [5]. TRP channels are capable of integrating multiple concomitant channel-activating stimuli (i.e., cold and menthol in the case of TRPM8) and amplifying these stimuli via calcium influx and membrane depolarization. Understanding the mechanisms by which thermosensitive TRP channel activities are regulated is likely to yield important insights into thermosensation.

Many membrane receptors and channels undergo regulated exocytosis to and endocytosis from the PM. Some examples include regulated translocation of AMPA, NMDA, and GABA receptors [6], [7], GLUT4 glucose transporters [8], CFTR and the ENaC epithelial sodium channel [9], and the Aquaporin 2 (AQP2) water channel [10]. Regulated exocytosis has also been reported to control TRP channel currents [11]. Insertion of vesicles containing TRPs into PM can alter current amplitude by regulating the number of functional channels on the cell surface as demonstrated for TRPV2 in response to IGF-I [12], TRPC5 after EGF stimulation [13], TRPV1 under NGF stimulation [14], [15], TRPM7 on muscle cells exposed to shear stress [16], TRPV5 with pH variations [17], and TRPA1 after treatments with mustard oil [18]. Confocal and TIRF microscopy indicate that the TRP channels TRPM1 [19], TRPML1 [20] and TRPM7 [21] are also contained in mobile punctae resembling vesicular structures, suggesting that the subcellular distribution of these channels may also be dynamically regulated.

Here we used single-particle tracking (SPT) to measure the movement of TRPM8-containing particles in transfected HEK-293T and F11 cells under TIRF microscopy. Our data demonstrate that punctae containing TRPM8 channels tagged with green fluorescent protein (TRPM8-EGFP) constitutively undergo distinct patterns of movement including rapid lateral movement in or very near the PM (i.e., within the evanescent field), and axial z movements into and out of the near field (i.e., exo- and endocytosis). Furthermore, the equilibrium between lateral and vertical movements is sensitive to the cholesterol-depleting agent methyl-beta-cyclodextrin (MβCD), which decreases vertical movement and stabilizes TRPM8-GFP fluorescence in the plasma membrane.

## Results

### TRPM8 particles are scattered near the PM

**Figure 1:**
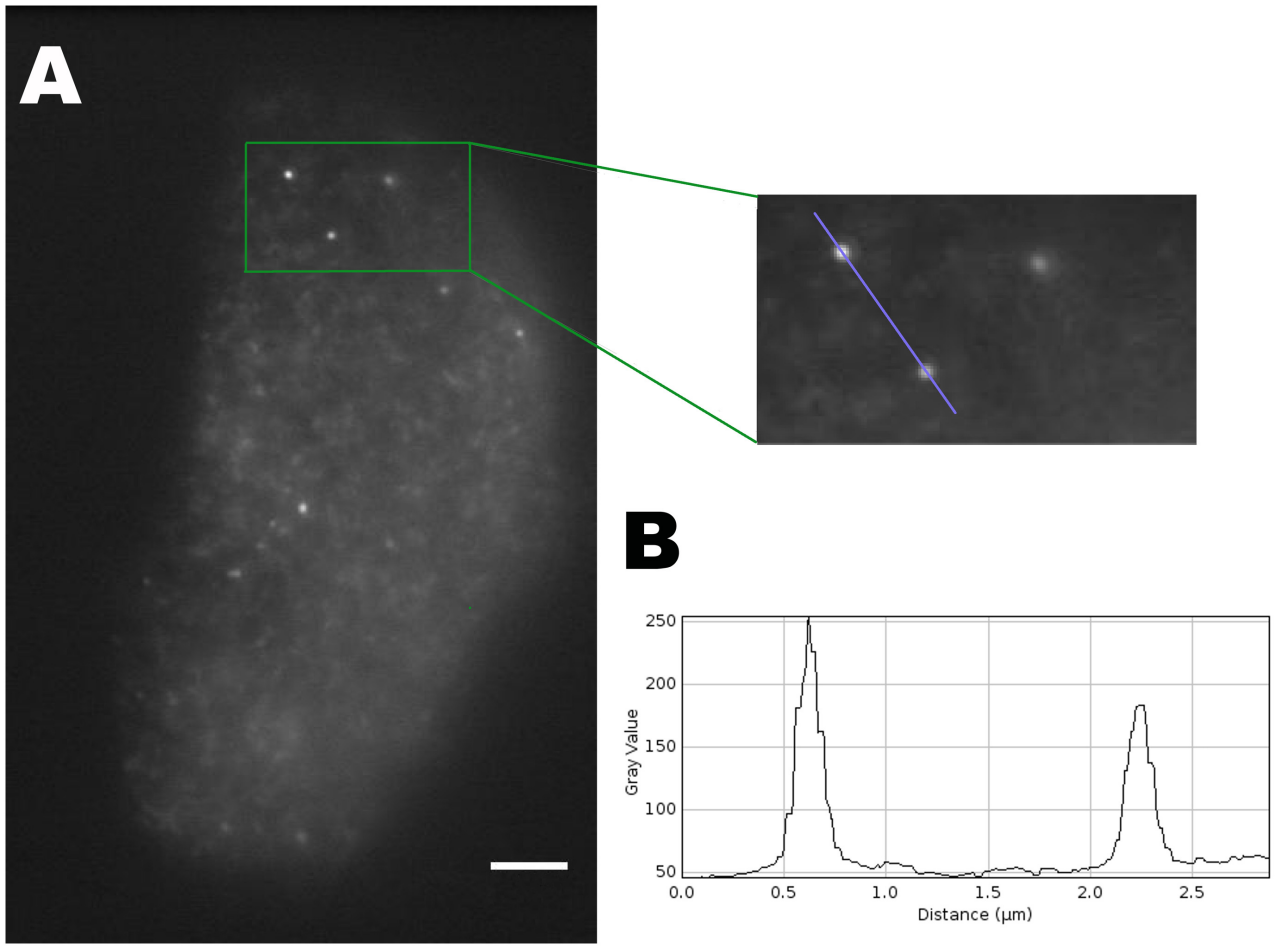
Particle imaging. ***A***, Image showing a TRPM8-GFP transfected F11 cell as an example for our low transfection conditions. Bar = 3 µm. *Inset*, magnified section of the cell showing three particles near the surface. ***B***, profile plot for the intensity of fluorescence over the blue line showed on the inset above.

Previous reports showed that different TRP channels have a characteristic mobile punctated pattern of expression at the level of the plasma membrane [13], [16], [17], [21]. However, TRP channel membrane dynamics have not been studied in detail. Consistent with previous reports for TRP channels in other cell types, TIRF microscopy revealed that TRPM8-EGFP fluorescence is primarily localized within punctae in both HEK-293T and F11 cells (Figure 1A). Under our conditions of low-level TRPM8 expression, we observed about 1 vesicle per µm^2^ in the evanescent field with a high signal to noise ratio (Figure 1B). The diameter of TRPM8-containing particles was 320±30 nm (n = 65; Figure 1B) and these particles did not exhibit any tendency to aggregate under resting conditions. Furthermore, fission and fusion of motile particles was rarely observed, and TRPM8-EGFP punctae appeared to move in a discrete network without manifesting any apparent preferred sites for vertical translocation.

### TRPM8 stoichiometry from single-spot bleaching

**Figure 2:**
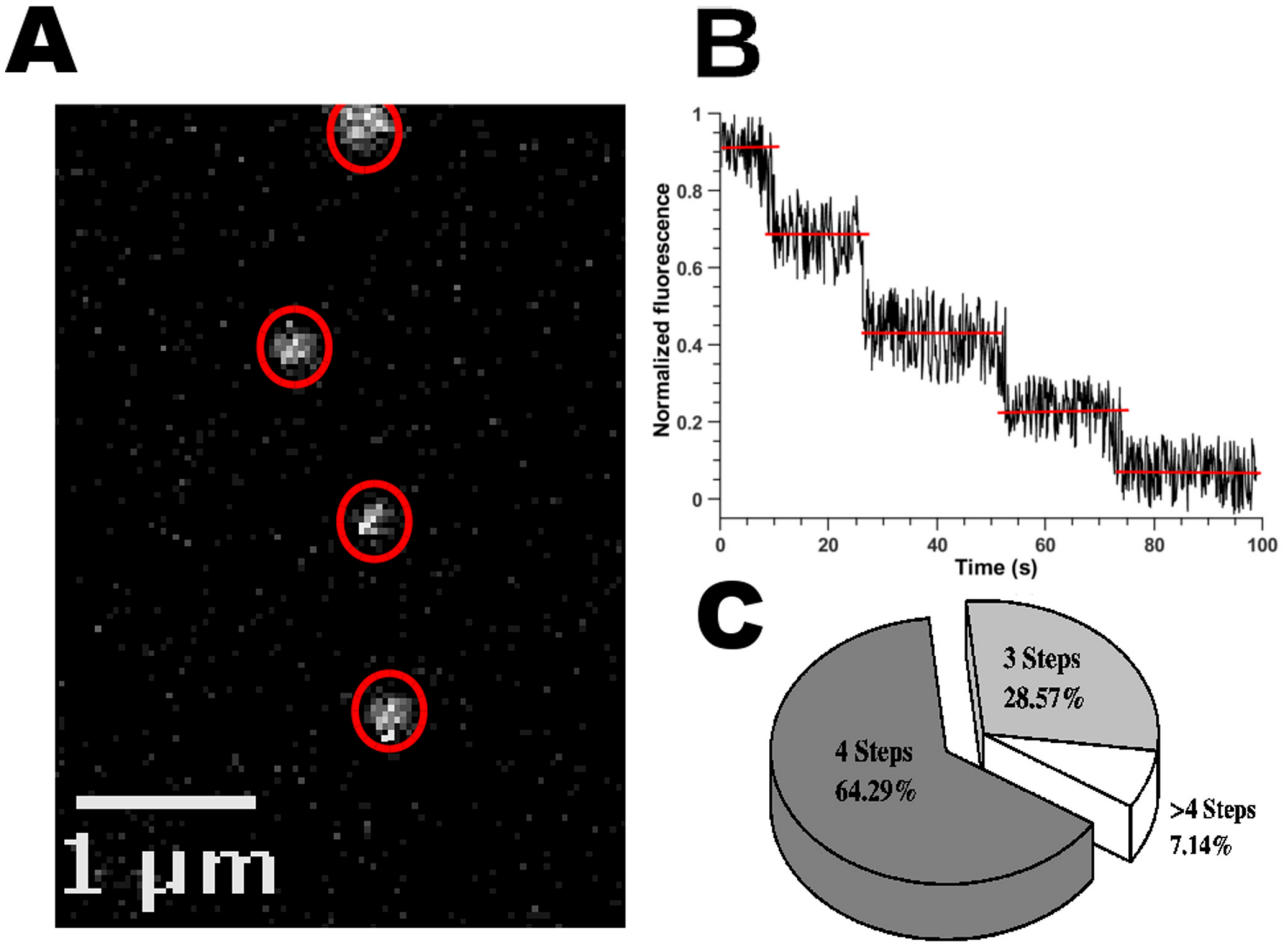
Particle stoichiometry. ***A***, TIRF image showing examples for the measured ROIs over bright spots selected for bleaching. ***B***, Intensity profile showing multiple-step loss of emission. For this particular example four bleaching steps are clearly discernible. ***C***, Pie plot showing the distribution for a total of 56 bleached spots. The more represented case is four steps of bleaching, however, 3 steps of bleaching are also highly represented. There are cases in which we observe more than four steps.

The consistent and small size of the observed TRPM8-EGFP punctae suggested to us that each fluorescent particle may correspond to a single vesicle containing a fixed number of TRPM8 channels. To determine the number of TRPM8 subunits present in each spot, we used a single-molecule photobleaching technique in which EGFP bleaching is stochastic and each EGFP molecule bleaches independently [22], [23]. Because each channel complex contains as many EGFP molecules as it does subunits, single-molecule photobleaching of EGFP fluorescence facilitates a direct count of the number of TRPM8 subunits present in any given bright spot. The rapid lateral movement of TRPM8-EGFP channels made it difficult to observe multiple bleaching events from the same particle, but incubation of cells in glucose-free media for 2 hr. slowed vesicle movement, enabling us to count the number of photobleaching steps in a number of vesicles (Figure 2 A and B). Because of their sequence similarity to the *Drosophila* Shaker potassium channel, a prototypical 6-transmembrane-spanning (6-TM) cation channel, TRP channels were initially assumed to adopt a tetrameric quaternary architecture [24]. Biochemical and atomic force microscopy (AFM) data support this prediction for TRPM8 channels [25], [26]. In order to estimate the number of channels present on each observed particle, we therefore assumed that TRPM8 channels are organized as tetramers.

Although the maximum number of bleaching steps that we observed was 8, the majority of the particles displayed only 4 bleaching steps (Figure 2C). The distribution of bleaching steps shows that a significant population of punctae undergoes only 3 steps of bleaching while others exhibit between 5 and 8 steps (Figure 2C). The bleaching profile observed in our TRPM8 samples was similar to that previously reported by Ulbrich and Isacoff (2007) for tetrameric CNG channels. As reported previously [23], we also observed many short, barely-distinguishably transitions. Our data therefore suggest that some of the EGFP molecules are either already bleached at the beginning of the recording, non-fluorescent (perhaps due to protein misfolding), or distant enough from the PM that the fluorescence signal-to-noise ratio was too low for accurate single-molecule resolution [23]. A reasonable and conservative conclusion from our experimental data is that TRPM8 is a tetramer, and that the particles we observe contain no more than 2 channel complexes.

### Classification of single-particle trajectories into different diffusion modes

**Figure 3:**
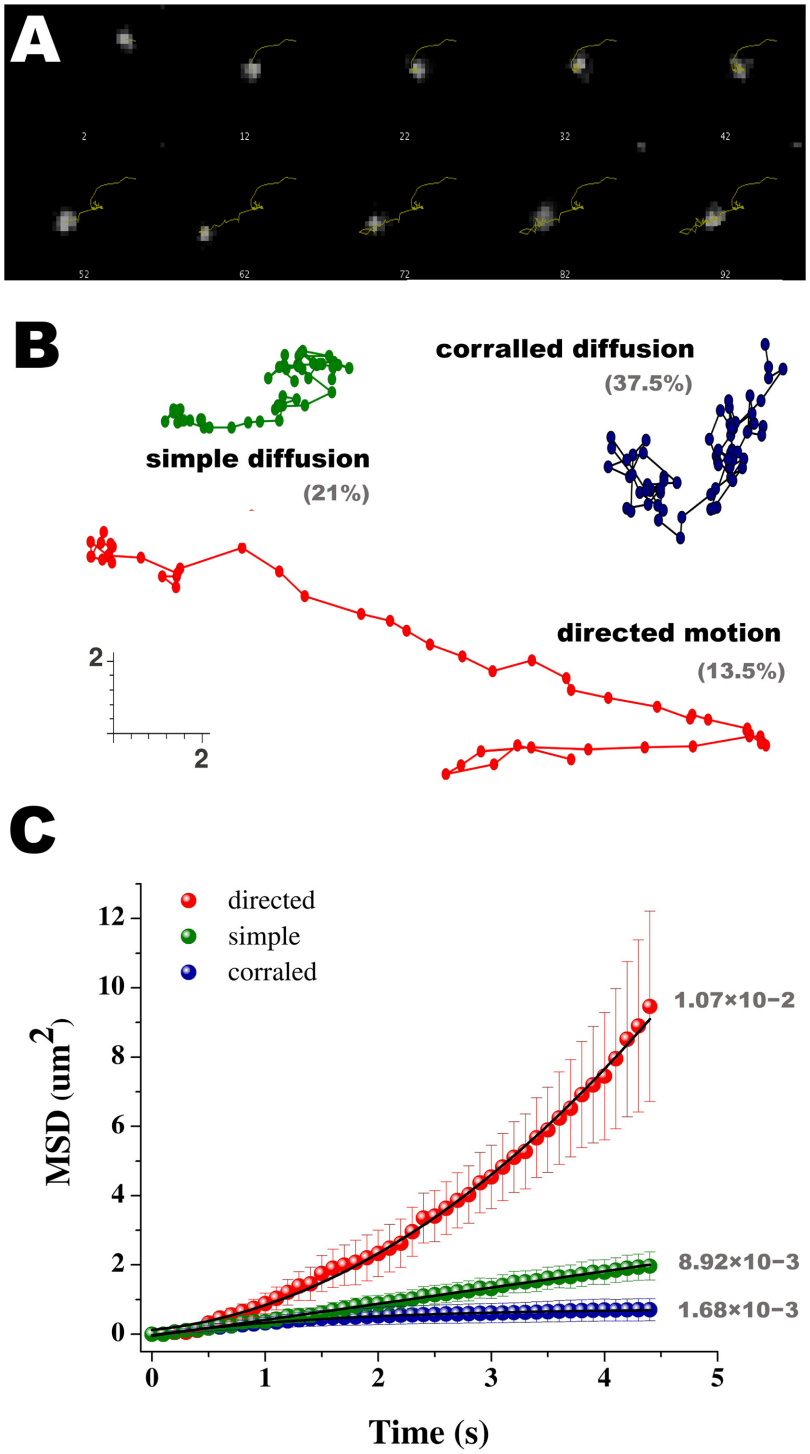
Modes of diffusion. ***A***, Montage of a selected 100 frames trajectory. ***B***, *S*elected 50-frame trajectories representing the different groups observed. Strict Brownian diffusion and anomalous diffusion were gathered in one single group (simple diffusion) in our study. The scale represent distance in µm. The distribution for the different diffusion modes is noted in percentage units. ***C***, Coordinates obtained from single particle tracking can be used to plot the mean square displacement (MSD) versus time interval. MSD vs time plot showing assembled averaged data for the three diffusing sub-groups observed. In this representation, the diffusion of individual particles can be classified into different modes, based upon fitting to the equations described on methods. n = 352 trajectories; error bars correspond to SD.

TIRF video microscopy demonstrated a high level of basal lateral trafficking of TRPM8-containing punctae in or near the PM in both HEK-293T and F11 cells (Movies S1 and S2). In contrast, TRPV3-EGFP particles are immobile in transfected cells (Movie S3), perhaps due to scaffolding by a type I PDZ-binding motif located at the C-terminus [27]. To quantify TRPM8 dynamics, we used STP to measure all moving vesicles in the focal plane of the TIRF image (Figure 3A). Fluctuations in vesicle fluorescence suggest that under resting conditions, TRPM8-containing particles tend to stop at defined places when they are close to the plasma membrane (as deducted from fluctuations in vesicle fluorescence; Movies S1 and S2). Once reaching maximal fluorescence, the vesicles appear confined to small domains (corrals) with a radius of 0.4–0.8µm (n = 24). Channel molecules reside within these corrals for 2–8 s (Figure 3B and 3C). Rarely, more than one fluorescent spot was observed to simultaneously stop at the same location. Particles often underwent long-range (∼10µm) lateral movements, presumably approaching the PM along the way (Figure 3B).

In order to describe the variety of different motions seen for TRPM8-containing particles, we perform a mode-of-motion analysis. Bright fluorescent spots exhibiting a diffusion coefficient that was indistinguishable from that of an immobilized probe on a glass coverslip was classified as immobile (D<1×10^−5^ µm^2^/s). The remaining particles were classified in three different groups according the goodness of fit to a plot of mean square displacement (MSD) vs. time: (a) simple Brownian diffusion; (b) directed motion, and (c) confined diffusion mode. The latter corresponds to particles that diffuse freely but are confined within a limited membrane area (Figure 3B).

To extract more information about the behavior of individual fluorescent particles, we measured each particle trajectory and, according to the initial selection criteria described above, classified each into static, simple Brownian, directed, or confined diffusional modes. After grouping, the corresponding MSD vs. time plots were averaged, and ultimately we were able to suitably fit each population to functions describing the different idealized diffusion behaviors (Figure 3C). The diffusion coefficients for simple, directed, and confined modes were 8.92×10^−3^ µm^2^s^−1^, 1.07×10^−2^ µm^2^s^−1^, and 1.68×10^−3^ µm^2^s^−1^ respectively. The size of the corrals obtained from curve fitting (0.75 µm on radius) correlates well with the values we obtained previously by measuring corrals directly.

### TRPM8 undergoes hop-diffusion

**Figure 4:**
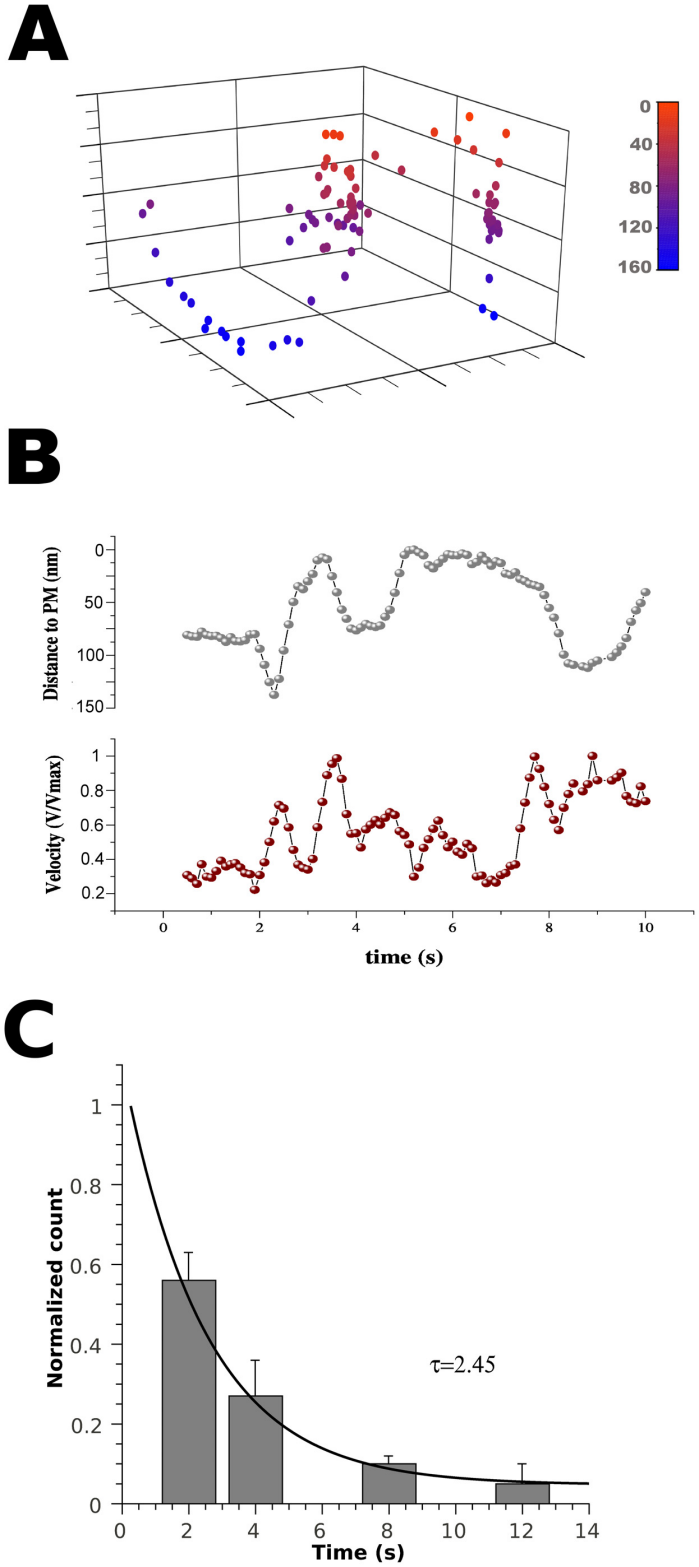
Diffusional description in 4D. ***A***, time sequence for a moving particle in three dimensional space (x,y,z) during 20 s. The z axis was calibrated using fluorescent beads of known size (see methods). Depicted particle goes from the inside of the cell (blue colors) to the plasma membrane (red colors), and jump from one corral to the next. The arrow indicates the starting point for the trajectory. ***B***, Smoothed plots for the same trajectory, showing the correlation between the position on the z axis (upper panel) with particles' change in velocity (lower panel). ***C***, Histogram for the residence time at the maximal fluorescence intensity. The solid line correspond to a first order exponential decay with an associated time constant indicated (n = 20).

Often vesicles approach or leave the evanescent field by moving perpendicular to the coverslip (Figure 4A). Constant axial movement of fluorescent particles, followed by changes in particle's mode-of-motion resembling hop-diffusion (particles “jumping” from one membrane corral to the next; Figure 4A) was quantified by measuring changes in fluorescence fluctuation that result from changes in the distance from the coverslip while simultaneously tracking vesicle velocity in the X-Y plane (Figure 4B). Our results demonstrate that when the z distance reaches its minimum (i.e., the particle is most closely associated with the membrane), diffusion in the X-Y axis also reaches a minimum value. Furthermore, we found that changes in axial position are correlated with a corresponding acceleration of the particle (Figure 4B). Analysis of 4-D plots showed that vesicles often approach the PM and stop just below the point of maximal fluorescence for a fraction of a second (∼5 frames or 500 ms), and then fluorescence continues to increase. We interpret these events to represent vesicle association with the PM. The time constant for the dwell time at the membrane was 2.5 s (Figure 4C), suggesting that vesicles reside in the PM for a few seconds before undergoing subsequent axial or X-Y movement. Particles that jump from one corral to the next helped us to determine that particle bleaching was minimal under our recording configuration (100 ms illumination, 20% laser power equivalent to 1.2 mW*s^−1^*µm^−1^) because particles maintained the same value of maximal fluorescence over a time span that includes multiple approaches to the PM.

### Cholesterol removal is sufficient to stabilize TRPM8 channels at the PM

**Figure 5:**
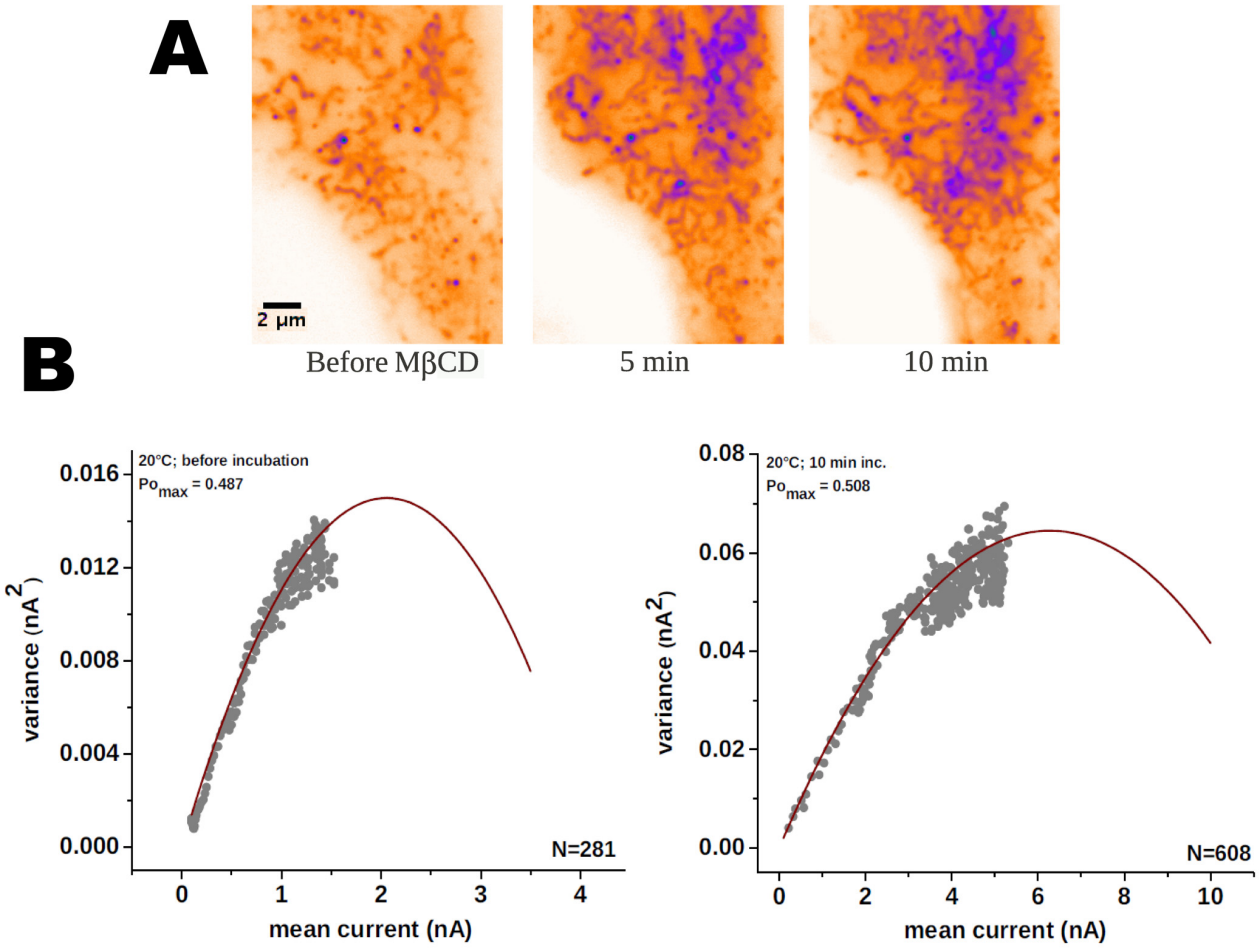
The effect of methyl-beta-cyclodextrin (MβCD). ***A***, Images representing overall activity after 200 frames (addition of all frames). Blue shade spots represent higher intensity, therefore an augment in vesicle residency over the recording. A 5 minutes treatment is enough to augment TRPM8-EGFP density at the plasma membrane. ***B***, Variance analysis made on HEK293 cells stable expressing TRPM8 channels showing an increase in the average current correlated with a three fold increment in the number of active channels at the membrane (n = 4).

Methyl-beta-cyclodextrin (MβCD) is often used as a tool to remove cholesterol from the PM. Cholesterol removal is considered an standard approach for lipid raft destabilization and recently was used to suggest that TRPM8 is located in lipid rafts [28]. According to the model of Morenilla-Palao, et al. [28], the transit of TRPM8 channels into and out of a cholesterol-rich microdomain can modulate channel activation, subsequently affecting the threshold for neuronal responses to thermal cooling. In contrast to our expectation, 5 mM MβCD treatment did not cause an increase in lateral motion in our samples (i.e., X-Y movement of brighter particles). Instead, particles accumulated into clusters in the evanescent field, close to the PM. This accumulation was evident after 5 min of incubation (Figure 5A). The augment in channel density makes impossible to perform an accurate mode-of-motion analysis. MβCD did not cause an evident increase in the PM fusion rate, suggesting that a decrease in the rate of steady-state vesicle endocytosis may account for the increase in TRPM8 fluorescence caused by MβCD. To address the question of whether MβCD altered the number of functional channel complexes in the PM, we recorded voltage-activated whole-cell TRPM8 currents at 20°C. In agreement with a previous report [28], and our optical measurements, after 10 min. incubation with 5 mM MβCD we observed an increase in the maximal current (I_max_) at +180 mV (Figure 5B). As I_max_ = i×N×P_(O)max_, where i is unitary current, N is the number of channels, and P_(O)max_ is the maximum open probability that can be achieved, a change in any of these parameters will cause maximal current amplitude to change. To address whether the treatment affects P_(O)max_, i, or N, we directly measured open probability at +180 mV using non-stationary noise analysis (see methods) before and after MβCD (5 mM) incubation. 10 minutes treatment caused the number of channels to increase 2–3 fold without affecting the P_(O)max_ or the unitary conductance (n = 4; Figure 5B).

## Discussion

SPT provides detailed information about the spatial and temporal dynamics of proteins near the plasma membrane, permitting direct estimation of microdomain compartment size and individual molecule residence time within these compartments. We observed the dynamics of single vesicles containing GFP-tagged TRPM8 channels using TIRF microscopy and characterized their diffusional modes. Under conditions of low expression in heterologous expression systems, TRPM8-containing vesicles are located near the PM and highly mobile. SPT analysis yielded diffusion constants that are about 1 order of magnitude faster than the values previously reported for the related TRPC5 (D∼5×10^−3^ µm^2^/s; [13]) and TRPV5 (D∼2 to 10×10^−4^ µm^2^/s; [17]) channels. However, the diffusion constants calculated for TRPC5 and TRPV5 were determined directly from the slope of the MSD vs time plots assuming simple Brownian diffusion. It is worth noticing that the value reported by Bezzerides et al. (2004) for TRPC5 is in good agreement with the value we obtained for simple Brownian motion of TRPM8-containing particles.

### The key may reside on lingering

At least 3 modes of vesicle diffusion have been proposed: full collapse, kiss-and-run, and kiss-and-linger [29]. Vesicles containing the glucose transporter 4 (GLUT4) exhibit full collapse fusion after insulin stimulation [30], and synaptic vesicles are known to undergo full collapse and kiss-and-run fusions [29], [31], [32]. In contrast, PM recruitment of TRPC5- and TRPV5-containing vesicles by stimulation with EGF or extracellular alkalinization, respectively, was characterized by longer membrane dwell times than would be expected for a kiss-and-run fusion, and therefore appear to kiss-and-linger [13], [17].

Mechanisms that stabilize the fused vesicle for longer times may allow channels to access to the extracellular solution via the lumen of the fused vesicle, thereby permitting vesicle-associated channels to contribute to membrane currents that are measured with whole-cell voltage clamp [13], [17]. Our data for TRPM8, where particles reside at the membrane up to few seconds confined within defined corrals, agree with a kiss-and-linger mechanism for channel insertion. This interpretation is supported by our observations that: a) TRPM8-containing vesicles change their diffusion mode without a decrease of the fluorescence signal after reaching the time of maximal fluorescence intensity; b) changes in fluorescence intensity and particle acceleration are correlated; c) particles retain integrity during their hop-diffusion. However, the mechanism of hop-diffusion/kiss-and-linger described here differs from the rapid translocation-insertion mechanism previously proposed by Bezzerides et al. (2004) for TRPC5, for which the rate of fusion was reported to increase. Such an increase in the rate of TRPM8-containing vesicle fusion was not evident in our experiments. Future studies, perhaps using antibody-conjugated quantum dots (Qdots) in combination with TIRF microscopy [33] in a native system (i.e. DRG neurons), will be required to determine whether the mechanisms controlling of TRPM8 channel residency on the PM are similar to those observed here in heterologous expression systems. We have failed so far into find a good extracellular antibody for labeling TRPM8 channels after Qdot conjugation.

### Altering PM lipid composition is sufficient to stabilize TRPM8-containing vesicles

Recently it was reported that TRPM8 is localized within cholesterol-rich lipid microdomains, suggesting that the transit of TRPM8 channels into and out of such PM microdomains may be a regulated process [28]. In good agreement with the report of Morenilla-Palao et al. (2009), we found that MβCD increases whole cell current without changing open channel probability, suggesting that vesicle fusion effectively increases the number of available channels. We failed to observe the expected lateral diffusion of TRPM8 channels at the PM [28], indicating that lateral movement into and out of lipid microdomains is unlikely under our experimental conditions. Our data therefore suggest a somewhat different mechanism for regulating TRPM8 channel availability. We propose that MβCD treatment alters basal vesicle trafficking by slowing the release of vesicles from PM, effectively tethering TRPM8 to the PM ultimately increasing channel density. Because we did not observe a change in the vesicle fusion rate, it seems unlikely that MβCD acts to recruit TRPM8 mobile vesicles that are near the PM.

**Figure 6:**
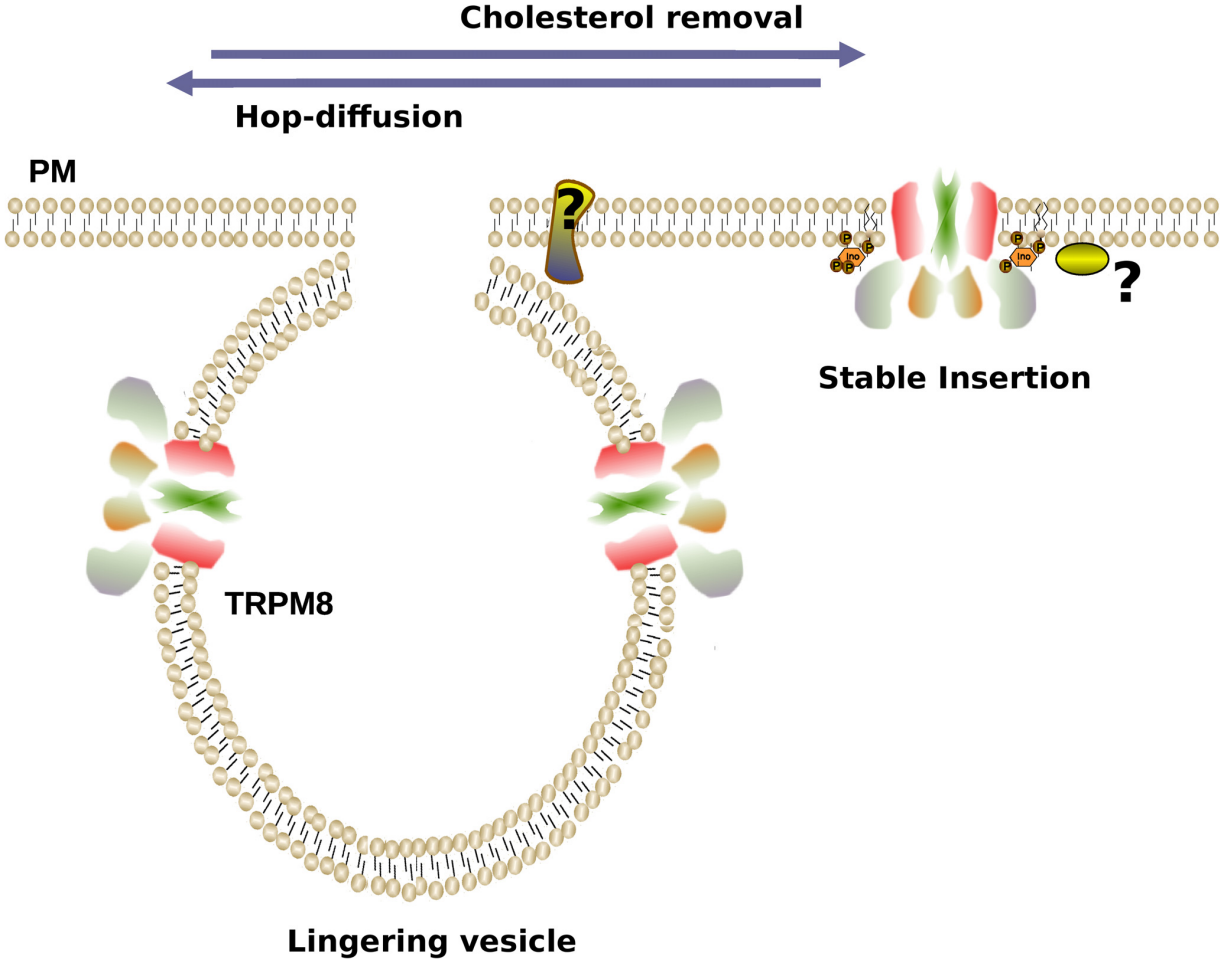
Model for hop-diffusion/stabilization of TRPM8 channels. According to our results an important population of TRPM8 channels undergo hop-diffusion. This behavior can be changed to extreme opposite conditions when membrane composition is altered. MβCD treatments are sufficient to stabilize the channels at the plasma membrane. We expect that hop diffusing vesicles containing TRPM8 channels are stabilized at the membrane, tethered but not fully fused, in a so called fuse-and-linger mode.

Figure 6 summarizes our model describing the effect of MβCD on TRPM8 exocytosis. According to the model, TRPM8-containing vesicles approach the PM and fuse but do not disassemble, resulting in lingering at the PM. Once docked at the PM, vesicle residency is subject to regulation by factors in the immediate environment, such as membrane lipids. Under control conditions, vesicles are not stabilized and continue to hop from one membrane corral to the next. In addition to the direct effect of MβCD to alter PM cholesterol content and thereby change membrane lipid microdomain composition and structure, the effect of MβCD on TRPM8-containing vesicles could also be mediated by other mechanisms [34], including clathrin-mediated endocytosis [35], [36] and cytoskeletal protein stability [37]. Further studies are needed to more precisely determine the mechanism of MβCD action and, more importantly, determine the effect of TRPM8 activators (i.e. menthol and temperature) on vesicle dynamics.

### Regulated exocytosis of TRPM8 channels and signal amplification

Some important questions that arise from our work are: a) Do TRPM8 channels normally mediate ionic conductance in intracellular (vesicle) membranes, and if so, what controls channel opening? b) Does the TRPM8 conductance affect the trafficking itinerary of vesicles in which the channels are transported? It is tempting to speculate that vesicle stabilization at the plasma membrane arise from a positive feedback loop by TRPM8-mediated Ca^2+^ flux, where immobile TRPM8 channels at the PM could be thought of as signal amplifiers. In our heterologous expression conditions, the population of TRPM8-containing vesicles is composed primarily of a static (∼35%) and a highly dynamic pool that transits between corrals (∼40%). If highly mobile population represents a reserve of TRPM8 channels that are “on-hold” and awaiting an appropriate locus or stimulus for PM insertion, adding these channels could dramatically increase the number of available channels and thereby provide a significant amount of potential signal amplification. Additional regulatory complexity could be provided if TRPM8 channel activity also controls the trafficking of TRPM8-containing vesicles.

Future studies of TRP channel movements using high resolution imaging and particle tracking techniques are likely to reveal surprising and important mechanisms for controlling the localization and activity of this diverse family of signaling proteins.

## Methods

### Molecular Biology

DNA coding for rat TRPM8 (kindly provided by Dr. D Julius, UCSF; GenBank accession number NM_134371) and human TRPV3 (kindly provided by Dr. David Clapham; GenBank accession number NM_134371) were used in this work. Channel coding sequences were cloned in frame into the EcoRI and XmaI sites of the mammalian expression vector pEGFP-N1 (Clontech Laboratories, Palo Alto,CA).

### Cell culture

HEK293T cells (Kindly donated by Dr. DE Clapham, HHIMI; [19]), were cultured in DMEM (Gibco Inc.) supplied with 10% BFS (Gibco Inc.). F-11 cells (Kindly donated by Dr. SE Gordon, U Washington; [15]) were cultured in F12 Ham's nutri-mix (Gibco Inc.) supplied with 20% BFS (Gibco Inc.) and HAT supplement mix (Sigma). Cells were plated in ploy-l-lysine coated coverslips and transfected using lipofectamine 2000 (Invitrogen,Inc.) according to the manufacturer's instructions. Recordings were made 24–36 hours after transfection. HEK-293 cells that stably express TRPM8 (CR#1 cells; [5]) were used in patch clamp experiments and were maintained in the presence of 600 µg/ml G418 until 24 hr prior the experiment.

### TIRF Microscopy

To selectively excite a thin layer of cytosol immediately adjacent to the coverslip, cells were imaged by using an objective-based TIRF microscope. 473-nm and 568-nm diode pump lasers (LaserGlow) were focused onto 3.5 µm single-mode optical fibers (Oz optics) and transmitted via the rear illumination port of an Olympus IX70 microscope. A digitally synchronized mechanical shutter (Vincent Associates) controlled exposure times. Laser light reflected from a dichroic mirror (Z488/532RPC; Semrock) passed through a high-numerical aperture objective (60×, N.A. 1.49, oil; Olympus) and was totally internally reflected by the glass–water interface. Fluorescence transmission was passed through a laser grade eGFP filter set (Semrock), and collected by a cooled-CCD (ORCA ER II; Hamamatsu) which has a pixel size of 67 nm×67 nm at the specimen plane. Under our experimental conditions, fluorescent objects were within 170 nm of the glass–water interface as measured using 6µm fluorescent beads according to the protocol described by Matthieses and Axelrod (2006) [38]. Measured X-Y plane resolution was 160 nm. The calculated error for X and Y axis were 0.67 and 0.78 nm respectively. GFP fluorescent images were acquired at 100-ms intervals (10Hz) using ImageJ (NIH) and the micro-manager plug-in (Universal Imaging). Imaging experiments were performed at 20C°.

### Tracking TRPM8 containing particles

We have employed a SPT plug-in for ImageJ (v1.42; http://rsb.info.nih.gov/ij/) called *ParticleTracker*, (Computational Biophysics Lab, ETH Zurich; http://www.cbl.ethz.ch/). *Particle Tracker* provide the X,Y coordinates for the center of detected bright spots (particles) and the average intensity for the all pixels contained in the particle's defined area. The original plug-in has been modified by us to add functionalities and make us able to calculate the Mean Square Displacement (MSD) for the obtained trajectories of near-membrane moving molecules and to automatize the whole analyzing procedures. The MSD was calculated for each particle at each frame n as follows:
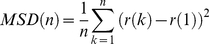
(1)where r(k) is the 2D position of particle in frame k. The diffusion constant (D) was calculated by the use of MSD vs. time plots [39]. Once the trajectories of membrane molecules and the plots of MSD vs time were obtained, they were fitted to the following equations:
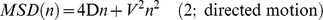


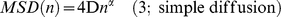


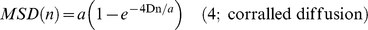



### Approximation to 3D movements

According to the exponential relationship between the fluorescence intensity of a particle and its vertical position as the particle moves within evanescent field, the vertical position of the particle can be obtained from the changes in vesicle's fluorescence intensity [38], [40]. Here we calculated vertical displacement as: zn = −d ln(Fn-1-Bn-1)/(Fn−Bn), where d is the penetration depth, Fn and Bn are the peak intensity and local background in frame n, respectively. Fluorescence from TIRF microscopy, has also a time-bleaching behavior which accounts for the loss of intensity on fluorescent granules. This time-decaying process is normally assumed to decrease exponentially in a very similar fashion than the distance dependent decay. However, because of eGFP stability, short exposition times (10–30 s) and low intensity incidental light, we can't detect significant bleaching on our samples, hence un-corrected images were used. Instantaneous 2D velocity was calculated as: V(t) = (r(t)−r(t−1))/Δt; where V(t) and r(t) are the instantaneous velocity and the particle's position at time t respectively, and Δt correspond to the acquisition time.

### Recording solutions and reagents

An external solution containing (in mM) 140 NaCl, 10 Hepes, 5 KCl, 2 MgCl2, 2 CaCl2, 10 Glucose, 295 mOsm, pH 7.4 was used. In some cases a divalent-free recording solution containing (in mM) 150 NaCl, 10 TRIS, 0.1 EGTA, 10 Glucose, 295 mOsm, pH 7.4. A cesium solution containing (in mM) 150 CsCl, 10 TRIS, 0.1 EGTA, 295 Osm, pH 7,4 was used for symmetrical ionic conditions on whole cell recordings. All reagents were obtained from Sigma-Aldrich.

### HEK293 Electrophysiology

Whole cell currents were obtained from HEK293 CR#1 cells. Gigaseals were formed using 2–4 MΩ borosilicate pipettes (1.5 mm OD, 0.86 mm ID, Warner Instruments Corp.) Whole cell voltage clamp was performed at 20C°. Macroscopic currents were acquired at 10 kHz and filtered at 4 kHz. Data acquisition was made using an Axopatch 200 (Axon Instruments), a 6052E acquisition board (National Instruments) and winWCP software (SIPBS, Strathclyde University, Glasgow, UK).

### Variance Analysis

Non-stationary noise analysis [41] was carried out at 20C°. To estimate the maximum probability of opening, we collected 50 current records during activation of the channels by a 150 ms depolarization voltage step from 0 to +180 mV. Ensemble averaged current, *<I>*, and its variance, σ^2^, on each isochrone were computed. The variance as a function of *<I>* data was fitted by the equation:
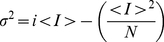
(5)where i is the single-channel unitary current and N the number of channels in the patch. The maximum open probability, P_omax_, was obtained according to the relation P_omax_ = I_max_/(iN) where I_max_ is the maximum mean current measured in the experiment. For noise analysis data was acquired at 20 kHz and filtered at 5 kHz.

### Data Analysis

Population averages were expressed as mean ± SEM. ANOVA and Bonferroni tests were used. We analyzed 20 independent experiments comprising 40 cells and a total of 520 particles to establish control conditions. Tracking analysis was performed as described above using our own routines written in Java. The statistical analysis was performed with GraphPad Prism 5.01 (GraphPad Software). Data plot analysis and curve-fitting were done with Origin 7 (Microcal Corp.). Figures were designed with The GIMP 2.2 (GNU, General Public License).

## Supporting Information

Movie S1 20s sequence on HEK-293T cells expressing TRPM8-GFP channels. Color palette was inverted for clarity.
(8.81 MB AVI)

Movie S2 20s sequence on F11 cells expressing TRPM8-GFP channels. Color palette was inverted for clarity.
(17.61 MB AVI)

Movie S3 40s sequence on F11 cells expressing TRPV3-GFP channels.
(44.79 MB AVI)

## References

[1] Clapham DE (2003). TRP channels as cellular sensors.. Nature.

[2] Ramsey IS, Delling M, Clapham DE (2006). An introduction to TRP channels.. Annual review of physiology.

[3] Dhaka A, Viswanath V, Patapoutian A (2006). TRP ion channels and temperature sensation.. Annu Rev Neurosci.

[4] Mckemy DD, Neuhausser WM, Julius D (2002). Identification of a cold receptor reveals a general role for TRP channels in thermosensation.. Nature.

[5] Brauchi S, Orio P, Latorre R (2004). Clues to understanding cold sensation: thermodynamics and electrophysiological analysis of the cold receptor TRPM8.. Proceedings of the National Academy of Sciences of the United States of America.

[6] Barry MF, Ziff EB (2002). Receptor trafficking and the plasticity of excitatory synapses.. Curr Opin Neurobiol.

[7] Triller a (2003). Synaptic structure and diffusion dynamics of synaptic receptors.. Biology of the Cell.

[8] Watson RT, Kanzaki M, Pessin JE (2004). Regulated membrane trafficking of the insulin-responsive glucose transporter 4 in adipocytes.. Endocr Rev.

[9] Peters KW, Qi J, Johnson JP, Watkins SC, Frizzell RA (2001). Role of snare proteins in CFTR and ENaC trafficking.. Pflugers Arch.

[10] Tamma G, Procino G, Strafino A, Bononi E, Meyer G (2007). Hypotonicity induces aquaporin-2 internalization and cytosol-to-membrane translocation of ICln in renal cells.. Endocrinology.

[11] Cayouette S, Boulay G (2007). Intracellular trafficking of TRP channels.. Cell calcium.

[12] Kanzaki M, Zhang YQ, Mashima H, Li L, Shibata H (1999). Translocation of a calcium-permeable cation channel induced by insulin-like growth factor-I.. Nature cell biology.

[13] Bezzerides VJ, Ramsey IS, Kotecha S, Greka A, Clapham DE (2004). Rapid vesicular translocation and insertion of TRP channels.. Nature cell biology.

[14] Zhang X, Huang J, Mcnaughton PA (2005). NGF rapidly increases membrane expression of TRPV1 heat-gated ion channels.. EMBO J.

[15] Stein AT, Ufret-Vincenty CA, Hua L, Santana LF, Gordon SE (2006). Phosphoinositide 3-kinase binds to TRPV1 and mediates NGF-stimulated TRPV1 trafficking to the plasma membrane.. J Gen Physiol.

[16] Oancea E, Wolfe JT, Clapham DE (2006). Functional TRPM7 channels accumulate at the plasma membrane in response to fluid flow.. Circ Res.

[17] Lambers TT, Oancea E, de Groot T, Topala CN, Hoenderop JG (2007). Extracellular pH dynamically controls cell surface delivery of functional TRPV5 channels.. Molecular and cellular biology.

[18] Schmidt M, Dubin AE, Petrus MJ, Earley TJ, Patapoutian A (2009). Nociceptive signals induce trafficking of TRPA1 to the plasma membrane.. Neuron.

[19] Oancea E, Vriens J, Brauchi S, Jun J, Splawski I (2009). TRPM1 forms ion channels associated with melanin content in melanocytes.. Science signaling.

[20] Dong X, Cheng X, Mills E, Delling M, Wang F (2008). The type IV mucolipidosis-associated protein TRPML1 is an endolysosomal iron release channel.. Nature.

[21] Brauchi S, Krapivinsky G, Krapivinsky L, Clapham DE (2008). TRPM7 facilitates cholinergic vesicle fusion with the plasma membrane.. Proceedings of the National Academy of Sciences of the United States of America.

[22] Iino R, Koyama I, Kusumi A (2001). Single molecule imaging of green fluorescent proteins in living cells: E-cadherin forms oligomers on the free cell surface.. Biophys J.

[23] Ulbrich MH, Isacoff EY (2007). Subunit counting in membrane-bound proteins.. Nat Methods.

[24] Latorre R, Zaelzer C, Brauchi S (2009). Structure-functional intimacies of transient receptor potential channels.. Quarterly reviews of biophysics.

[25] Dragoni I, Guida E, McIntyre P (2006). The cold and menthol receptor TRPM8 contains a functionally important double cysteine motif.. J Biol Chem.

[26] Stewart AP, Egressy K, Lim A, Edwardson JM (2010). AFM imaging reveals the tetrameric structure of the TRPM8 channel.. Biochem Biophys Res Commun.

[27] Xu HX, Ramsey IS, Kotecha SA, Moran MM, Chong JHA (2002). TRPV3 is a calcium-permeable temperature-sensitive cation channel.. Nature.

[28] Morenilla-Palao C, Pertusa M, Meseguer V, Cabedo H, Viana F (2009). Lipid raft segregation modulates TRPM8 channel activity.. The Journal of biological chemistry.

[29] Ryan TA (2003). The life and times of a neurosecretory granule..

[30] Lizunov VA, Matsumoto H, Zimmerberg J, Cushman SW, Frolov VA (2005). Insulin stimulates the halting, tethering, and fusion of mobile GLUT4 vesicles in rat adipose cells.. The Journal of cell biology.

[31] Sudhof TC (2004). The synaptic vesicle cycle.. Annual review of neuroscience.

[32] Zhang Q, Li Y, Tsien RW (2009). The dynamic control of kiss-and-run and vesicular reuse probed with single nanoparticles.. Science.

[33] Chang Y, Pinaud F, Antelman J, Weiss S (2008). Tracking bio-molecules in live cells using quantum dots.. Journal of biophotonics.

[34] Munro S (2003). Lipid rafts: elusive or illusive?. Cell.

[35] Rodal SK, Skretting G, Garred O, Vilhardt F, van Deurs B (1999). Extraction of cholesterol with methyl-beta-cyclodextrin perturbs formation of clathrin-coated endocytic vesicles.. Molecular biology of the cell.

[36] Subtil A, Gaidarov I, Kobylarz K, Lampson MA, Keen JH (1999). Acute cholesterol depletion inhibits clathrin-coated pit budding.. Proc Natl Acad Sci U S A.

[37] Kwik J, Boyle S, Fooksman D, Margolis L, Sheetz MP (2003). Membrane cholesterol, lateral mobility, and the phosphatidylinositol 4,5-bisphosphate-dependent organization of cell actin.. Proc Natl Acad Sci U S A.

[38] Mattheyses AL, Axelrod D (2006). Direct measurement of the evanescent field profile produced by objective-based total internal reflection fluorescence.. Journal of biomedical optics.

[39] Saxton MJ, Jacobson K (1997). Single-particle tracking: applications to membrane dynamics.. Annu Rev Biophys Biomol Struct.

[40] Axelrod D, Burghardt TP, Thompson NL (1984). Total internal reflection fluorescence.. Annual review of biophysics and bioengineering.

[41] Alvarez O, Gonzalez C, Latorre R (2002). Counting channels: a tutorial guide on ion channel fluctuation analysis.. Advances in physiology education.

